# Bioaccumulation of gamma emitting radionuclides in *Polysiphonia fucoides*

**DOI:** 10.1007/s10967-013-2794-2

**Published:** 2013-10-23

**Authors:** Tamara Zalewska

**Affiliations:** Institute of Meteorology and Water Management, National Research Institute, Maritime Branch in Gdynia, Waszyngtona 42, 81-342 Gdynia, Poland

**Keywords:** Marine macroalgae, Radionuclides, Bioaccumulation

## Abstract

The article presents the results of a study on the bioaccumulation abilities of *Polysiphonia fucoides*, a red algae specific to the southern Baltic Sea, towards (of) gamma emitting isotopes. A laboratory experiment was carried out to determine changes in the activities of some isotopes—^54^Mn, ^57^Co, ^65^Zn, ^110m^Ag,^113^Sn, ^134^Cs, ^137^Cs and ^241^Am—occurring in *P. fucoides* exposed to a seawater medium containing these isotopes over the course of 1 month. All analyzed isotopes showed the greatest increase of radioactive activity in plant tissue in the first 24 h of exposure. The temporary concentration factors of cesium isotopes were increasing linearly during the experiment from 114 to 274 in the case of ^137^Cs, and from 144 to 351 in the case of ^134^Cs. The level of the initial concentration factor of cesium isotopes in the plant proved to be independent of the initial concentration of the isotope in seawater and it took the lowest (125 dm^3^ kg^−1^) level among the studied isotopes. In the case of a mixture of gamma emitting isotopes, a linear relation between the individual isotope activity in *P. fucoides* and its initial concentration in seawater was established after the first day of exposure; the isotopes initial concentration factors ranged from 767 to 874 dm^3^ kg^−1^. Having reached the maximal concentration level, a statistically significant decline in radioactivity concentrations of the five isotopes in the plant tissue was observed. A half-life of biological removal of the isotopes from the plant tissue was established at: 3.8 days in the case of ^54^Mn, 4 days—^57^Co, 4 days—^60^Co, 4.2 days—^137^Cs and ^241^Am—3.5 days.

## Introduction

Unequivocal characterization of the biosorption and bioaccumulation processes of radionuclides and heavy metals by macroalgal species under environmental conditions is a difficult task because of the complexity of the processes per se, as well as due to the numerous influencing factors [5–7, 23]. The differentiation between those processes is already a predicament. Biosorption is a simple physiochemical process resembling conventional adsorption or ion exchange [4]. Simultaneously, biosorption is the initial stage of bioaccumulation, defined as intracellular accumulation. The understanding of the mechanisms and the mathematical characterization of the bioaccumulation processes of radionuclides and heavy metals by aquatic plants would assist the development of variable applications of this ability in environmental contexts [4, 6, 7, 21, 22].

Studies conducted on the bioaccumulation process of ^137^Cs and other radionuclides and heavy metals in marine plants have pointed to their considerable bioaccumulation ability irrespective of the area of the study: in the Black Sea [16–18, 20], Agean Sea [1, 11–13], New Caledonia lagoon [8], Northern Pacific—the Aleutian Chain [3], Mediterranean Sea [2].

Macroalgae are used mainly as bioindicators in environmental status assessments regarding contamination with hazardous substances, including heavy metals and radionuclides [2, 3, 8, 15, 20]. The main properties that are determinant in the wide application of aquatic plants as bioindicators are their common occurrence and relatively facile accessibility. An important factor is the exchange method of elements with the environment—the foliar uptake in the case of plants with the exclusion of the gastric tract, which makes the resultant interpretation difficult in the case of fauna. The foliar uptake also determines the fact that the contaminant concentrations detected in the plant tissue directly reflect changes occurring in the environment. Therefore, the response time to environmental changes is much shortened also because the intensive physiological processes and growth of the macroalgae takes place in a relatively short period of the year and thus are followed by increased uptake and quick response to contamination [10].

Studies on macroalgae bioaccumulation and bioindication processes carried out in the Baltic Sea area [14, 19, 26, 28–30] have indicated that taking into account plant morphology, e.g. comparing green algae, red algae, brown algae and vascular plants, the key element in bioaccumulation efficiency is the construction of the thallus. Filamentous algae or plants with an expanded exterior, which facilitates the exchange of elements with the environment, are at an advantage. *Polysiphonia fucoides*, a red algae, proved to be one of the best accumulating plants and contained the highest activity concentrations of ^137^Cs in consecutive years, and the highest concentration coefficient values [26, 28, 29]. Its bioaccumulation ability towards other radioactive isotopes was confirmed in preliminary laboratory experiments [27].

The primary goal of the presented study was the determination of the abilities of *P. fucoides* to accumulate cesium isotopes, which are still of considerable concern within the Baltic Sea area because of the increased level of ^137^Cs [25]. The bioaccumulative abilities were also examined towards a mixture of gamma emitting isotopes (^54^Mn, ^57^Co, ^65^Zn, ^109^Cd, ^110m^Ag, ^113^Sn, ^137^Cs and ^241^Am), some of which are released from nuclear power plants in routine operational procedures. The appropriate designation of experiments carried out in 2010 and in 2011 made it possible to follow the bioaccumulation process via changes in isotope activities in the plant tissue and seawater medium. The experiment with a single isotope exposure followed by one with a mixture of radioactive elements indicated the effect of competitive isotope accumulation on that of cesium ions.

## Materials and methods

### General outline of the experiment

**Fig. 1:**
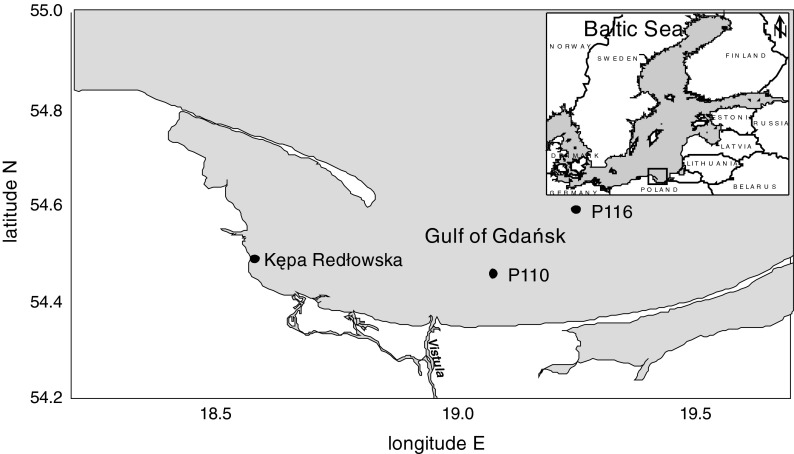
Sampling location

The bioaccumulation process of gamma emitting radionuclides was examined in the red algae *P. fucoides* under laboratory conditions in two separate experiments carried out in 2010 and 2011. Macrophytes were sampled in the area of Kępa Redłowska, in the Gulf of Gdańsk (Fig. 1). The plants were collected with the stony substrate by scuba divers on 5th October 2010 and on 28th April 2011. The stones covered with red macroalgae were rinsed with seawater, in order to remove sand, solid pollutants and organisms (e.g. *Gammarus* spp.) living in the thalli. The stones were transported to the laboratory submerged in the seawater. The plants were immersed in aquariums of 50 × 80 × 50 cm dimensions, equipped with aerating filters. The aquariums were filled with local seawater. Water temperature was adjusted to ambient temperature and was 20 ± 1 °C, while water salinity was about 7.0 (PSS’78).

In 2010, the plants were placed in three aquariums and left to equilibrate for 1 day. On the next day (6th October 2010), 1 and 2 ml of standard ^137^Cs solution (code JM/25/O/00, activity 21 Bq g^−1^/09.11.2000; produced by the OBRI POLATOM, Poland) were added to two of the aquariums, A1 and A2 respectively. To the third aquarium (A3), 2 ml of standard ^134^Cs solution (code BW/Z-63/14/10, total activity 1.37 ± 0.02 kBq g^−1^/01.05.2010, total weight 10.14714 g; produced by the OBRI POLATOM, Poland) was added. Because of the intensive mixing of water in the aquariums by pressurized air, the isotope concentrations homogenized immediately. At this point, samples for the determination of initial isotope concentrations were taken.

In 2011, the plants were placed in one aquarium only and left to equilibrate for 2 days. On the third day (30th April 2011), 1 ml of mix gamma standard solution (code BW/Z-62/27/09, total activity 72.67 kBq/15.06.2009, total weight 10.02732 g; produced by the OBRI POLATOM, Poland) was added to the aquarium. The standard solution contained a mixture of 11 radionuclides (^51^Cr, ^54^Mn, ^57^Co, ^60^Co, ^65^Zn, ^85^Sr, ^109^Cd, ^110m^Ag, ^113^Sn, ^137^Cs, ^241^Am). The initial concentrations of radionuclides in the spiked seawater were calculated using certificated values and seawater volume.

The sampling of plant material was done in the same manner on both experimental occasions, in 2010 and 2011. The first sampling was done 1 day after the spiking, and the subsequent samplings were done every day until the fifth day of the experiment, and then every 3 days.

In 2010, in the experiment on ^134^Cs and ^137^Cs concentration changes, seawater samples from the aquariums were collected in parallel to plant tissue. Relatively high isotope concentrations in the aquariums made it possible to collect samples of very small volume—30 ml, therefore the conditions of the experiment were considered constant. Water samples were collected at the same temporal intervals as the plant material—every day on the first 5 days of the experiment and later every 3 days.

In the case of the experiment carried out in 2011, the concentrations of gamma emitting isotopes were below their detection limits in samples of 30 ml volume. The removal of samples of bigger volume (e.g. 450 ml), after the initial one, could not be continued as it caused considerable loss of seawater from the aquarium.

Initial environmental concentrations of radionuclides were determined in *P. fucoides* in specially designated samples collected at the same time as the plants for the exposure experiment. The concentrations of all analyzed radionuclides in environmental plant samples, except for ^137^Cs, were below their detection limits. The limit of detection values (expressed in Bq kg^−1^ DW) of the analyzed radionuclides in algae tissue were as follows: ^51^Cr—64.6, ^54^Mn—7.3, ^57^Co—4.8, ^60^Co—7.9, ^65^Zn—15.2, ^85^Sr—7.9, ^109^Cd—93.0, ^110m^Ag—6.1, ^113^Sn—7.6, ^137^Cs—6.8 and ^241^Am—22.8. Seawater samples of 450 ml volume were taken in situ parallel to the plants samples and radionuclide concentrations were measured in Marinelli geometry with the same gamma spectrometric method. Most radionuclide activities in environmental seawater were below their detection limits of ^51^Cr—0.82; ^54^Mn—0.08; ^57^Co—0.09; ^60^Co—0.11; ^65^Zn—0.15; ^85^Sr—0.9; ^109^Cd—2.04; ^110m^Ag—0.13; ^113^Sn—0.13; ^137^Cs—0.07; ^241^Am—0.28 Bq dm^−3^. The environmental concentrations of ^137^Cs in seawater were determined from monitoring data as^137^Cs is measured regularly in the seawater of the Polish sector of the Baltic Sea every year since 1984. Because cesium activity concentrations are quite uniform in the water column, the environmental concentrations were determined as a mean from the results of measurements at two stations in the Gulf of Gdańsk—P110 and P116 (Fig. 1) in 2010 and in 2011.

### Analysis

The macroalgae samples taken from the aquariums were dried and weighed to determine dry mass content, ashed at 450 °C and homogenized. Then, they were placed in cylindrical dishes of 40 mm diameter, and in such a form they were ready for radioactivity measurements.

The activity of seawater samples of 30 ml volume was measured in vessels of the same geometry as used for plant tissue.

The measurement of gamma emitting radionuclide activity was carried out with the gamma spectrometric method, using two spectrometric systems: an HPGe detector, with a relative efficiency of 18 % and a resolution of 1.8 keV for a peak of 1,332 keV of ^60^Co and an HPGe detector, with a relative efficiency of 40 % and a resolution of 1.8 keV for a peak of 1,332 keV of ^60^ Co. The detectors were coupled to an 8,192-channel computer analyzer. The analyses of radioactivity spectra, registered in 8,000 channels, was carried out by the Genie-2000 software. The measurement calibration was done using a gamma standard solutions mix produced by the OBRI POLATOM, Poland (code BW/Z-62/27/07).

The reliability and accuracy of the applied method was verified by participation in the HELCOM–MORS Proficiency Test Determination of Radionuclides in Fish Flesh Samples, organized by IAEA-MEL Monaco (IAEA-414, Irish and North Sea Fish). Fish muscle material may be recognized as a substitute for the ashed macroalgae samples because of nearly the same density of the prepared samples. The results of ^137^Cs and ^40^K determination in the test material are presented in Table 1 after IAEA report [9].

**Table 1:** Results of HELCOM–MORS proficiency test determination of radionuclides in fish flesh (IAEA 414—certified reference material)

Analyte	IAEA value (Bq kg^−1^ DW)	IAEA uncertainty (Bq kg^−1^ DW)	Laboratory value (Bq kg^−1^ DW)	Laboratory uncertainty (Bq kg^−1^ DW)	Laboratory uncertainty (%)	Accuracy (%)	Precision (%)
^40^K	481	16	474.5	19.3	4.1	1.35	5.2
^137^Cs	5.18	0.10	5.06	0.64	12.6	2.32	12.8

Laboratory No. 4, Institute of Meteorology and Water Management, Maritime Branch, Gdynia, Poland, Reference Date: 01 January 1997 modified after [9]

## Results and discussion

### Bioaccumulation of ^134^Cs and ^137^Cs

**Fig. 2:**
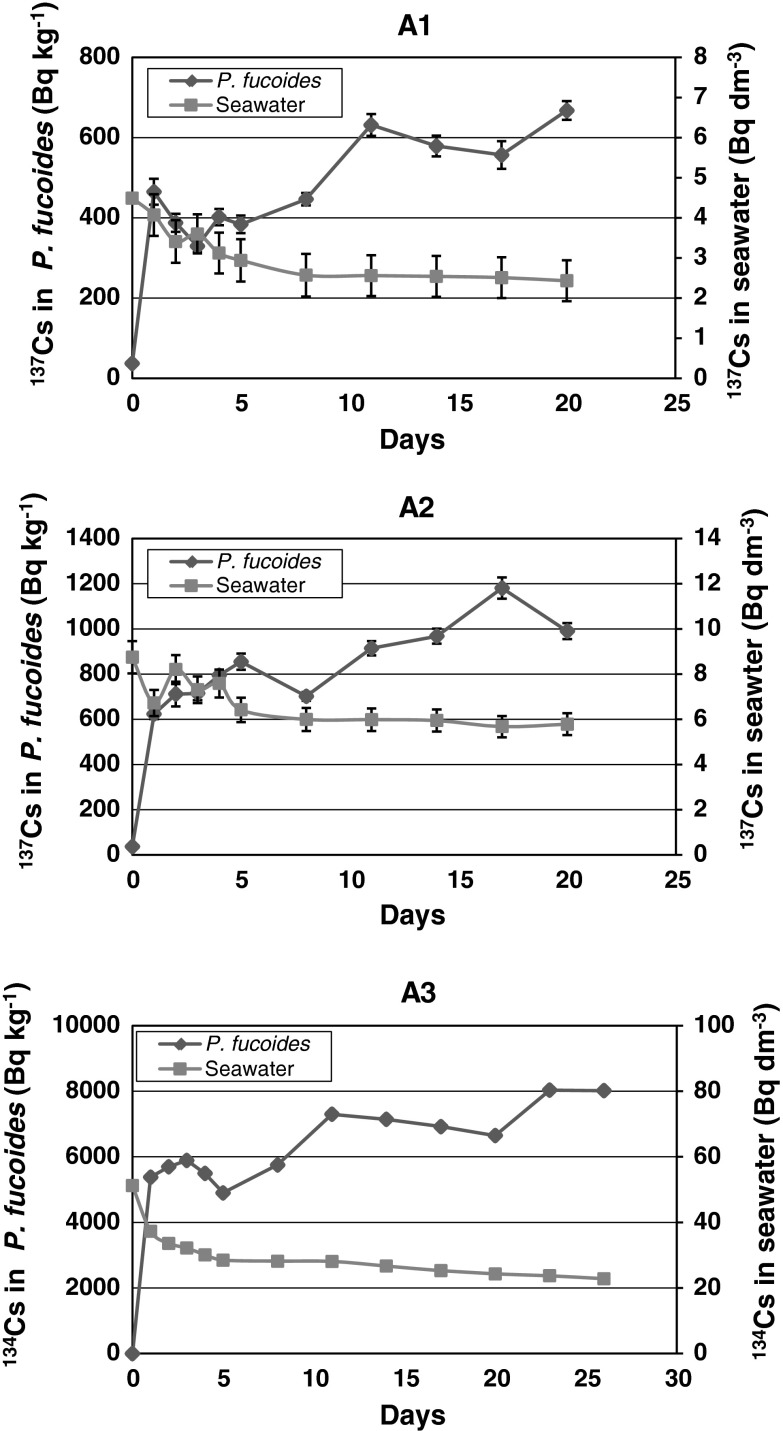
Changes of ^137^Cs and ^134^Cs activities in *P. fucoides* and seawater with exposure time

The maximal increase of ^134^Cs and ^137^Cs activity in *P. fucoides* was observed after the first day of exposure, independent of the initial radionuclide concentration in the medium (Fig. 2). The initial concentration of ^137^Cs (137-ceasium) in seawater was 4.49 **±** 0.13 Bq dm^−3^ in aquarium A1 and 8.75 **±** 0.18 Bq dm^−3^ in aquarium A2, while that of ^134^Cs in aquarium A3 was 51.3 **±** 0.3 Bq dm^−3^. Hence, the lowest experimental concentration of ^137^Cs (in A1) was already 160 times higher than the environmental concentration, 28.0 **±** 0.8 Bq m^−3^, determined in monitoring measurements in the Gulf of Gdańsk.

Activity concentrations of ^137^Cs in *P. fucoides* reached 465.0 ± 32.2 Bq kg^−1^ DW (dry weight) and 623.8 **±** 22.8 Bq kg^−1^DW in aquariums A1 and A2, respectively. Activity concentration of ^134^Cs in *P. fucoides* in aquarium A3 reached 5,381 ± 47 Bq kg^−1^ DW. The initial extremely intensive increase of radioactivity was probably related to the uptake of the radionuclides from the medium, occurring spontaneously and independently of metabolism and requiring no energy input [10]. Then other mechanisms of adsorption and transportation, both passive and active, may play a more important role. To be adsorbed, each ion has to pass barriers such as the laminar layer, the cell wall and the plasmalemma, before finally reaching the cytoplasm [10]. The thickness of the laminar layer depends on the turbulence occurring in the surrounding water. Under laboratory conditions, due to aeration, the effect of this layer can probably be excluded and the uptake will not be limited by the rate of diffusion across this layer. Generally, during the first stage, ions are introduced to the so-called apparent free space that, in seaweeds, includes the cell wall and all intercellular spaces exterior to the plasmalemma [10]. The apparent free space consists of two parts; the first of these is called the water free space and the second one, which relates to the deeper parts of the thallus, is the Donnan free space. Ions introduced to the water free space can be readily removed and subsequently reintroduced. This mechanism is likely to explain the oscillations in isotope activities observed in the first 5 days of exposure—ion transport in both directions until the equilibrium is reached in the system. The increase in isotope concentrations in the plant tissue on the 8th day of exposure, i.e. after the quasi-equilibrium was reached, could indicate an increase in the contribution of the active transport mechanism in the bioaccumulation process, the mechanism mainly linked to the plant metabolism. The bioaccumulation process at this stage is much less intensive because the plasmalemma might be much more difficult to penetrate unlike the cell wall which does not generally present a barrier to ion entry [10].

**Fig. 3:**
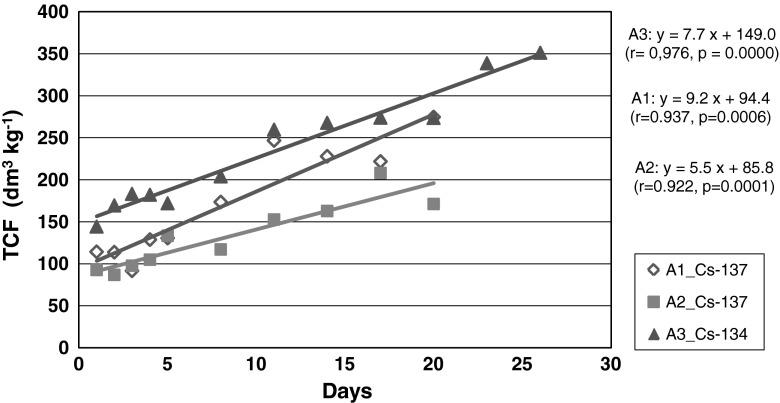
Changes of TCF with exposure time

The development of isotope activity changes in a seawater medium, illustrated by the curves in (Fig. 2), and is well reflected in the changes occurring in the plant tissue. The significant decrease in isotope activity concentrations took place during the first 5 days of exposure and, after the 8th day, the concentrations changed very little (Fig. 2). The concentration process of the ions in plant tissue is related to the dynamic steady state between the uptake and excretion processes. The concentration factor (CF) is the quantitative measure that allows us to assess the relative ability of biota representatives to absorb and/or take up the analyzed trace element. To follow the progress in bioaccumulation process, temporary concentration factors (TCF) were determined. The TCF of cesium isotopes were increasing over the entire experiment period; in the case of ^137^Cs—from 114 to 275 dm^3^ kg^−1^ in the aquarium A1 and from 93 to 207 dm^3^ kg^−1^in the aquarium A2. The range of TCF in the case of ^134^Cs was 144–351 dm^3^ kg^−1^. The increase of TCF values followed a statistically significant linear relationship (Fig. 3); *r* = 0.937 (*p* = 0.0006) in A1 aquarium, *r* = 0.922 (*p* = 0.0001) in A2 aquarium and *r* = 0.976 (*p* = 0.0000) in A3 aquarium.

The slope of the line, corresponding to the TCF daily increment (∆TCF), can be considered a rate of isotope bioaccumulation by *P. fucoides*. The calculated ∆TCF results were quite close in the case of ^137^Cs in aquarium A1 (8.5 dm^3^ kg^−1^ day^−1^) and ^134^Cs in aquarium A3 (7.7 dm^3^ kg^−1^ day^−1^). A slightly lower rate, 5.5 dm^3^ kg^−1^ day^−1^, was obtained in A2 aquarium for ^137^Cs.

Because the substantial change in activity was observed on the first day of exposure, initial concentration factors (CF_0_) were determined to compare bioaccumulation efficiency of *P. fucoides* towards a single isotope in this decisive stage of experiment; the CF_0_ was defined as the ratio of the isotope concentration in the plant tissue after the first day of exposure to its initial concentration in the seawater medium.

In the case of cesium isotopes—^137^Cs (in aquarium A1) and ^134^Cs (in aquarium A3)—CF_0_ values were nearly identical: 104 ± 7 and 105 ± 1 dm^3^ kg^−1^. This provided evidence that the CF_0_ did not depend on the initial isotope concentration in the seawater medium. The bioaccumulation process of ^137^Cs in A2 aquarium showed slightly different characteristics; the CF_0_ = 71 ± 6 dm^3^ kg^−1^ was much lower than in A1 and A3, and the changes of TCF in consecutive days varied resulting in a lower daily ∆TCF. The observed discrepancies indicate a different experimental regime, e.g. a much bigger plant biomass in the aquarium A2.

### Bioaccumulation of gamma emitting isotopes: ^54^Mn, ^57^Co, ^60^Co, ^65^Zn, ^109^Cd, ^110m^Ag, ^113^Sn, ^137^Cs and ^241^Am

**Fig. 4:**
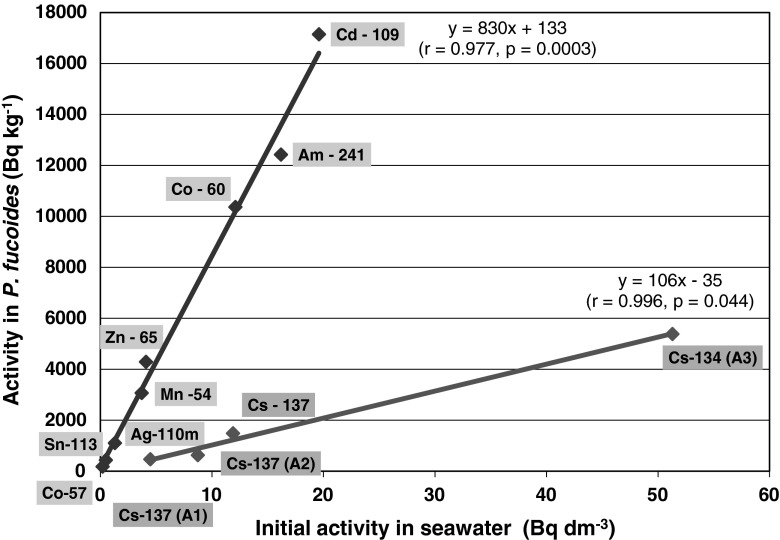
Relation of radionuclides activities in *P. fucoides* to the initial activities in seawater

**Fig. 5:**
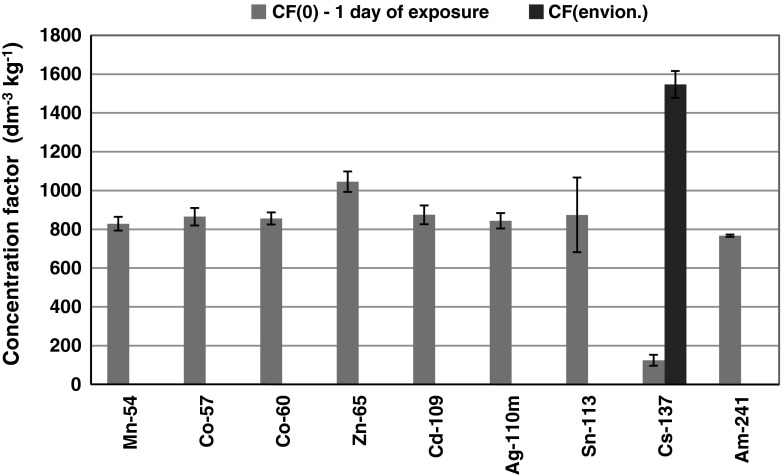
CF_0_ determined for radionuclides for first day of exposure

The experiment on the bioaccumulation of a mixture of gamma emitting radioisotopes by *P. fucoides* under laboratory conditions showed a statistically significant linear relation (*r* = 0.977, *p* = 0.0003) of the activity of isotopes in the plant tissue and their initial concentration in a seawater medium after the first day of exposure (Fig. 4; Table 2). ^137^Cs was an exception as its activity in *P. fucoides* was definitely lower than would be expected from the linear relationship. At the same time the concentration of cesium in the plant tissue was increasing proportionally to the concentrations in seawater (Fig. 4). However, the efficiency of cesium bioaccumulation was nearly eight times lower at the stage than in the case of other radioisotopes, well illustrated by the low slope of the correlation curve (Fig. 4). This low bioaccumulation efficiency at the initial stage was also marked in the low values of initial concentration coefficients CF_0_. In the case of ^137^Cs, CF_0_ = 135 ± 7 dm^3^ kg^−1^ was slightly higher than the value obtained in 2010. However, in the experiment in 2011, the concentration coefficient determined at the equilibrium stage reached 1,547 ± 69 dm^3^ kg^−1^, i.e. it was over 100 times higher. The CF_0_ characterizing the remaining isotopes were varying in a relatively narrow range 862–949 dm^3^ kg^−1^ in the case of ^54^Mn, ^57^Co, ^60^Co, ^109^Cd and ^110m^Ag (Fig. 5). ^65^Zn was marked for the highest CF_0_ = 1,125 ± 57 dm^3^ kg^−1^.

**Table 2:** Parameters characterizing bioaccumulation and depuration of gamma emitting radionuclides

	^54^Mn	^57^Co	^60^Co	^65^Zn	^109^Cd	^110m^Ag	^113^Sn	^137^Cs	^241^Am
Initial activity in seawater (Bq dm^−3^)	3.7 ± 0.1	0.50 ± 0.02	12.1 ± 0.4	4.1 ± 0.1	19.6 ± 0.6	1.26 ± 0.04	0.18 ± 0.01	11.9 ± 0.4	16.2 ± 0.6
Activity in *P. fucoides* after 1 day of exposure (Bq kg^−1^ DW)	3,066 ± 75	433 ± 17	10,359 ± 107	4,285 ± 155	17,139 ± 743	1,097 ± 34	175 ± 38	1,441 ± 50	12,421 ± 155
Maximum activity in *P. fucoides* (Bq kg^−1^ DW)	4,961 ± 94	819 ± 21	13,728 ± 130	7,767 ± 189	47,832 ± 638	1,846 ± 43	320 ± 44	1,465 ± 54	23,632 ± 207
Time of the maximum reaching (day)	5	5	5	8	8	8	5	5	5
Isotope bioaccumulation rate for the maximum reaching (Bq kg^−1^ DW day^−1^)	992 ± 19	164 ± 4	2,746 ± 26	971 ± 24	5,979 ± 80	231 ± 5	64 ± 9	284 ± 10	4,726 ± 41
Statistical significance of trends after reaching a maximum									
Correlation coefficient (*r*)	−0.934	−0.937	−0.918	0.690	0.098	0.296	–	−0.778	−0.912
Significance level (*p*)	0.0002	0.0019	0.0005	0.0398	0.8019	0.4391	–	0.0135	0.0006
Biological depuration rate constant (day^−1^)	0.044	0.037	0.035	–	–	–	–	0.029	0.060
Biological half time (day)	3.8	4.0	4.0	–	–	–	–	4.2	3.5

**Fig. 6:**
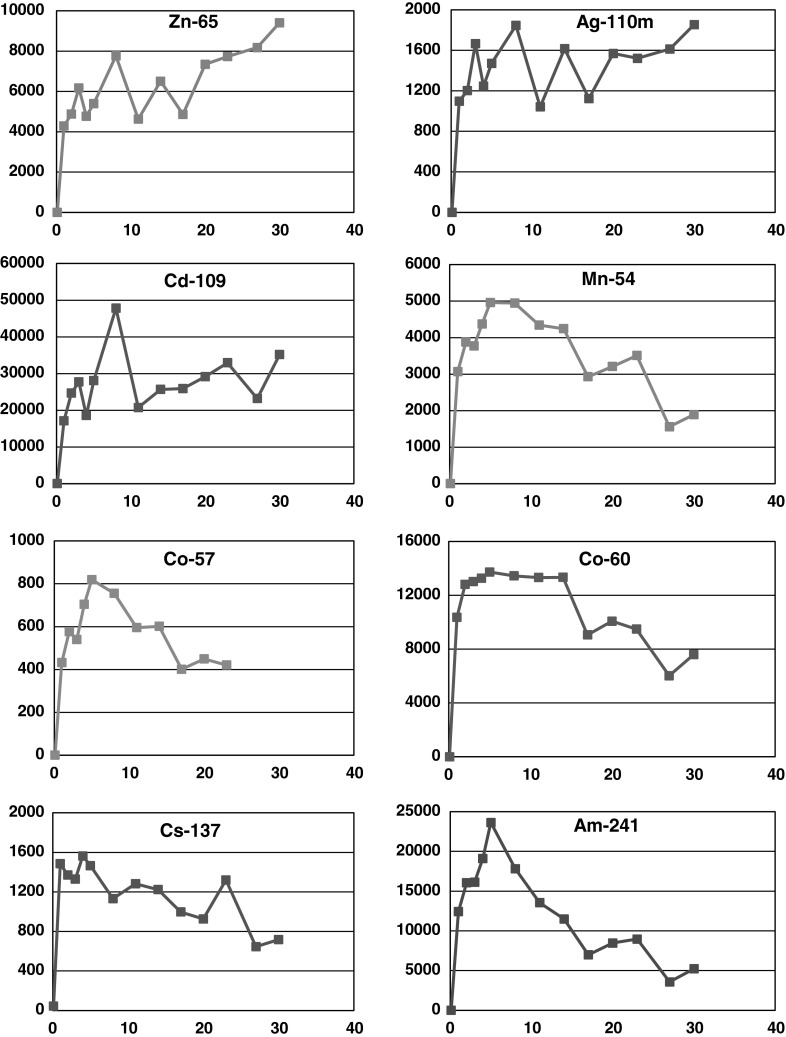
Changes of radionuclides activities (Bq kg^−1^ DW) in *P. fucoides* with time (days)

In the 2011 experiment, the initial development of the curves illustrating activity changes of individual isotopes in the plant tissues was very similar to that registered in the case of cesium isotopes in 2010 (Fig. 6). The extreme increase in activity concentrations was recorded on the first day of exposure, followed by further accumulation up to a maximum. The maximal level was reached by six isotopes in 5 days of exposure (Table 2). There were only three isotopes—^65^Zn, ^109^Cd and ^110m^Ag—whose activity reached the maximum in 8 days of exposure (Table 2). Further development of the bioaccumulation curves of ^65^Zn and ^110m^Ag differed from the other isotopes (Fig. 6). Beyond the maximum, in the subsequent days of the exposure experiment, their activity concentrations oscillated around a certain average level, which seemed to indicate that the bioaccumulation process had attained the quasi equilibrium stage. However, after 20 days, their activity concentrations again showed a slight increase. This might be the expression of the increased contribution of the active bioaccumulation in the overall bioaccumulation process, requiring an additional energy supply related to metabolic processes in the plant tissue. This observation confirms the greater importance of Zn and Ag as micronutrients in plant physiology. In the case of five radioisotopes—^54^Mn, ^57^Co, ^60^Co, ^137^Cs and ^110m^Ag, a statistically significant decrease in activity concentrations in *P. fucoides* was observed beyond the maximum (Fig. 6; Table 2). The decline in the isotopes activity could be related to the removal of the excess ion, especially facile in the case of ions adsorbed on the surface or taken out from the free space. The initial, extremely intensive bioaccumulation of ions from the seawater medium caused a considerable decrease in their concentrations in seawater, and this could be the reason for the more effective removal of ions from the plant thalli as an aftermath.

It worth noticing that the half-life time of the individual isotopes radioactive decay was of negligible importance taking into account the duration of the experiment. It played a meaningful role solely for ^113^Sn. Its activities in *P. fucoides* had already declined below the method detection limit after 11 days of exposure, mainly because of its low initial concentration in the seawater medium, and also due to its rather short half-life, ca. 115 days.

The results obtained in this decreasing, final stage of the experiment were applied to calculate the biological depuration rate constants (Table 2) in a single-component model described by equation [24]:

$$ A_{\text{t}} \; = \;A_{0} {\text{e}}^{{ - \lambda {\text{t}}}} , $$

where *A*_t_ is the activity of radionuclide at the end of experiment (Bq kg^−1^ DW), *A*_0_ is the maximum activity of radionuclide (Bq kg^−1^ DW), λ is the biological depuration rate constant (day^−1^), t is the time from the maximum until the end of experiment (day).

The constant λ allows the calculation of the radiotracer biological half-life according to the equation:

$$ T_{{{\raise0.7ex\hbox{${\text{b1}}$} \!\mathord{\left/ {\vphantom {{\text{b1}} {\text{s}}}}\right.\kern-0pt} \!\lower0.7ex\hbox{${\text{2}}$}}}} \; = \;\ln {\raise0.7ex\hbox{$2$} \!\mathord{\left/ {\vphantom {2 \lambda }}\right.\kern-0pt} \!\lower0.7ex\hbox{$\lambda $}}, $$

where *T*_b1/2_ is the biological half-life (day).

The determined biological depuration times of the analyzed isotopes were comparable (Table 2). Americium was the most easily removed isotope (*T*_b1/2_ = 3.5 days) and cesium ions were the hardest to eliminate (*T*_b1/2_ = 4.2 days). In two isotopes of cobalt, the determined biological depuration half-times were equal and reached 4 days.

The different forms of cesium isotopes bioaccumulation curves, in the part subsequent to maximum in a single isotope exposure experiment in 2010 and in a mixture exposure in 2011, could be the result of competitiveness of sorption and/or bioaccumulation. In a medium where no competing ions occur to block the active sorption locations and fulfill the free space, the activity concentration of cesium ions also increased subsequently to maximum. When other isotopes were present in the medium, characterized by decidedly higher bioaccumulation rates, cesium ions became removed from the plant tissue in the second stage of the experiment. One of the reasons for much slower bioaccumulation of cesium isotopes in comparison to other ions could be its oxidation status; it was the only ion with the first oxidation number—Cs^+^. This might affect the sorption to active spots in the outside plane and walls of the *P. fucoides* thalli. Cesium exhibited the largest ion diameter (c.a twice as large as other isotopes), which could hamper the passive and active transport into the plant tissue.

## Conclusions

- Bioaccumulation of cesium radioactive isotopes as well as other gamma emitting isotopes in P. fuoides under laboratory conditions showed similar features. The spectacular change in activity concentrations of the isotopes in *P. fucoides* was recorded in the first day of exposure to a seawater medium spiked with radioactive ions.
- Biosorption connected to surface binding of ions and bioaccumulation in the so-called free space, including the cell wall and all the cellular spaces exterior to the plasmolemma, are likely to be the dominant processes at this stage.
- During the entire experiment, the TCF of ^137^Cs and ^134^Cs increased linearly and their initial concentration coefficients CF_0_ did not depend on the initial isotope concentration in the seawater medium.
- The experiment on bioaccumulation of a mixture of gamma emitting radioisotopes by *P. fucoides* under laboratory conditions showed a statistically significant linear relation between the activity of isotopes in the plant tissue and their initial concentration in a seawater medium after the first day of exposure. Since this relation, in the case of ^137^Cs, was described by another equation, it provides evidence of different characteristics of the bioaccumulation process regarding radioactive cesium.
- Because of the very short time of environmental response and considerable bioaccumulation efficiency, *P. fucoides* should be recommended as a bioindicator of radioactive pollution, both under static and accidental circumstances.
